# Comparative study on sound production in different Holocentridae species

**DOI:** 10.1186/1742-9994-8-12

**Published:** 2011-05-24

**Authors:** Eric Parmentier, Pierre Vandewalle, Christophe Brié, Laura Dinraths, David Lecchini

**Affiliations:** 1 Laboratoire de Morphologie Fonctionnelle et Evolutive, Institut de Chimie, Bât. B6C, Université de Liège, B-4000 Liège, Belgium; 2 Tropical Fish Tahiti, Avatoru, 98729 Rangiroa, French Polynesia BP A5 - 98848 Noumea, New Caledonia; 3 CRIOBE, USR 3278 - CNRS / EPHE, Centre de Recherches Insulaires et Observatoire de l'Environnement, CBETM - Université de Perpignan, Moorea, French Polynesia

**Keywords:** Holocentridae, swimbladder, acoustic, biogeography, sounds, sonic muscle, Beryciform

## Abstract

**Background**

Holocentrids (squirrelfish and soldierfish) are vocal reef fishes whose calls and sound-producing mechanisms have been studied in some species only. The present study aims to compare sound-producing mechanisms in different Holocentridae genera (*Holocentrus*, *Myripristis*, *Neoniphon*, *Sargocentron*) from separate regions and, in some cases, at different developmental stages. An accurate comparison was made by recording six species while being hand-held, by observing TEM) the sonic muscles and by dissections of the sound-producing mechanism.

**Results**

In all these species, calls presented harmonics, their dominant frequency was between 80 and 130 Hz and they were composed of trains of 4 to 11 pulses with gradual increasing periods towards the end of the call. In each case, the calls did not provide reliable information on fish size. The sounds were produced by homologous fast-contracting sonic muscles that insert on articulated ribs whose proximal heads are integrated into the swimbladder: each pulse is the result of the back and forth movements of the ribs. Small differences in the shape of the oscillograms of the different species could be related to the number of ribs that are involved in the sound-producing mechanism. These fish species are able to make sounds as soon as they settle on the reef, when they are 40 days old. Comparison between *Neoniphon *from Madagascar and from Rangiroa in French Polynesia showed a new, unexpected kind of dialect involving differences at the level of pulse distribution. *Neoniphon *calls were characterised by a single pulse that was isolated at the beginning of the remaining train in Madagascar whereas they did not show any isolated single pulses at the beginning of the call in Rangiroa.

**Conclusion**

This family cannot use the acoustic fundamental frequencies (or pulse periods) of grunts to infer the size of partners. Pulse duration and number of pulses are statistically related to fish size. However, these characteristics are poorly informative because the correlation slope values are weak. It remains other features (sound amplitude, resistance to muscle fatigue, calling frequency) could be used to assess the body size. Characteristics of the sound producing mechanisms are conservative. All species possess fast-contracting muscles and have the same kind of sound producing mechanism. They do show some change between clades but these differences are not important enough to deeply modify the waveforms of the calls. In this case, our description of the grunt could be considered as the signature for the holocentrid family and can be used in passive acoustic monitoring.

## Background

Holocentrids are well known reef-dwellers with nocturnal habits. The family contains two sub-families [1]: 1) the Myripristinae or soldierfish with five genera (*Myripristis*, *Plectrypops*, *Corniger*, *Ostichthys *and *Pristilepis*) and 2) the Holocentrinae or squirrelfish with three genera (*Holocentrus*, *Neoniphon *and *Sargocentron*). Some fish in this family have quickly become of interest for their acoustic abilities because comparative anatomy and hearing experiments have clearly shown how the morphology and position of the swimbladder can influence hearing [2]. Different kinds of sounds (growls, knocks, grunts, staccato, thumps, growls) have been recorded in only a few species, such as in some *Holocentrus *[3-5] and *Myripristis *[6,7]. In *Holocentrus ascensionis*, "grunt" sounds were produced by residents when defending a crevice. The presence of a larger fish or a predator caused "staccato" sounds, accompanied by retreat into the crevice [4]. In *Myripristis *species (*M. berndti*, *M. amaena*, *M. violaceus*, *M. pralinius*), sounds were generally associated to two behaviours. Growls, thumps and knock were produced during episodes of chasing between conspecific in schools. Growls, grunts and staccato were reponse by field populations of *M. violaceus *to disturbances caused by a diver and or predators [6,7]. Unfortunately, the lack of homogeneity employed by different authors in the sound descriptions and terminology complicates comparisons.

In *Holocentrus rufus*, deeper analysis relating to muscle ablation [8] and physiology [9] showed that sound production results from the contraction of paired bilateral muscles inserting on the skull and on the first ribs in relation to the anterior part of the swimbladder. The contraction rate of the muscles depends on the firing rate of the motor axons innervating the sonic musculature and this determines the fundamental frequency (ca. 75-85 Hz) of the sound in this species [9].

The aim of the present study was to make a detailed analysis of the acoustic features of holocentrids by investigating the sounds and morphology in different species within the family. The aim was to discover how small modifications in morphology might influence the calls in this taxa. Sounds of different species from the three main genera *(Myripristis, Neoniphon *and *Sargocentron*) were recorded when the fishes were hand-held. The advantage of this approach was that all the fish were placed in the same behavioural condition and at the same distance from the hydrophone. Moreover, sounds were recorded from different regions (Madagascar and Rangiroa in French Polynesia) and it was also possible to record for the first time sounds in settling larval fish.

## Materials and methods

The first group of holocentrid specimens (21 *Sargocentron diadema*, *9 Myripristis kuntee *and 23 *Neoniphon sammara*) were collected in September and October 2007 by scuba diving in the coral reef area near Tulear (Mozambique canal, west coast of Madagascar) at depths of between 2 and 20 m. A solution of rotenone or a solution of quinaldine was used to catch the fish [10]. Fish were stored in two community tanks (3.5 × 0.7 × 0.2 m) with running seawater (26°C). Each tank was further divided into four smaller compartments. Rocks were provided to allow the fish to shelter.

*Myripristis violacea *were collected in May 2008. These specimens were at the larval stage and were caught with a net during the night when they settled on the reef crest. The net similar to the one used by Lo - Yat [11] was situated in a "hoa" (a small channel between the ocean and a lagoon). Adult *Myripristis violacea*, *Nenoniphon samara *and *Sargocentron spiniferum *were also caught during low tide in the hoa. Quinadlin was used to anaesthetise and to find fish hidden behind rocks. Fish were distributed between different tanks (0.7 × 1.4 × 0.4 m) with running seawater (27°C).

For both sites, sounds were recorded with an Orca hydrophone (sensitivity: -186 dB re 1 V/µPa). This system has a flat frequency response range (±3 dB) between 10 Hz and 23.8 kHz. The hydrophone was connected via an Orca amplifier (ORCA Instrumentation, France) to a Tascam recorder (TASCAM HD-P2). The hydrophone was placed in the centre of the tank (3.50 × 0.7 × 0.2 m in Madagascar and 0.7 × 1.4 × 0.4 m in Rangiroa). All fish were recorded in the same way. The fish was hand-held at a distance of 5 cm from the hydrophone, with the dorsal and pectoral fins blocked. About 30 sounds were recorded for each fish.

Sounds were digitised at 44.1 kHz (16-bit resolution) and analysed using AvisSoft-SAS Lab Pro 4.33 software [12]. Only the sounds with a high signal to noise ratio were used in the analysis. Temporal features were measured manually from oscillograms, and frequency parameters were obtained from power spectra (FFT size: 30 Hz). The sound parameters measured were: sound duration (ms); number of pulses in a sound; pulse period (measured as the average peak-to-peak interval between consecutive pulses in the entire sound, ms); interpulse interval (IPI, measured as the time from the end of one pulse to the beginning of the next, ms); pulse length (measured as the time from the beginning of one pulse to its end, ms); dominant (or main) frequency), which represents the most intense frequency (in Hz). In *Myripristis violacea*, the calling amplitude was measured from power spectra (referenced to the RMS amplitude).

In each species, five to eight specimens that had previously made sounds were euthanised by overdose immersion in MS-222. A specimen of each species was then rapidly dissected in order to expose the sonic muscles. Small samples of the sonic and epaxial muscle (1 cm^3^) were taken from four specimens and fixed in glutaraldehyde 2.5% for transmission electron microscopy (TEM). The other specimens were fixed in formaldehyde 5%. Two specimens of each species were later coloured with Alizarin according to the Taylor and Van Dyke method [13] in order to visualise osseous structures. These prepared specimens together with intact fishes, were dissected and examined with a Wild M10 (Leica Camera) binocular microscope equipped with a camera lucida. After glutaraldehyde fixation, muscle samples were dehydrated in an ethanol-propylene oxide series and were then embedded in epoxy resin (SPI-PON 812). The cellular ultrastructure was examined on ultrathin sections (60-80 nm) stained with uranyl acetate and lead citrate. The sections were viewed with a JEOL JEM 100SX transmission electron microscope under an 80-kV accelerating voltage.

Sounds and sound-producing mechanisms have been described in *Holocentrus rufus *[8,14]. However, in order to be certain of making a correct comparison between the different holocentrids, two *Holocentrus rufus *were obtained from a petshop in Belgium. These fish were recorded while being hand-held, and were then anesthetised, euthanised and dissected for comparison with previous descriptions and with other holocentrids in this study.

In the results, "n" refers to the total number of analysed sounds and "N" to the number of fishes; n = y, N = x means that the analysis was made on y sounds coming from x fishes. A t-test (non parametric test of Mann-Withney) was used to compare data between Polynesian and Madagascan *Neoniphon*, and between larval and adult *M. violacea*. Pearson correlation test was used to assess the relationship between the fish size and the different sonic characteristics.

Experiments were performed under control of the ethical commission of the University of Liège (form 07-728).

## Results

### Sounds

**Figure 1:**
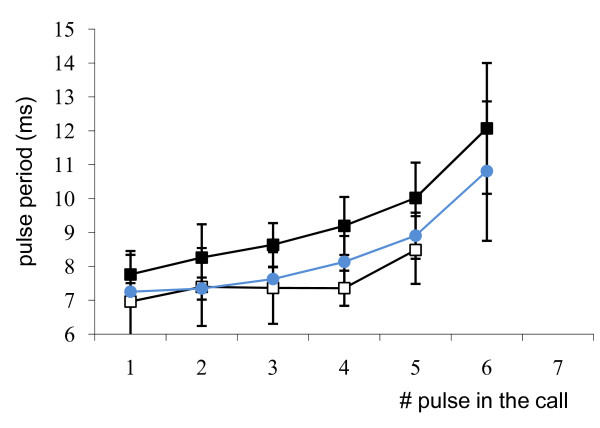
**Means of the successive pulse periods in calls of seven pulses in *Myripristis violacea *adults (Black Square), in *Myripristis violacea *larvae at settlement (White Square) and in *Neoniphon sammara *(Black Circle)**.

All species presented some common characteristics. The calls were all composed of a variable number of pulses with gradually increasing periods towards the end of the call (Figure 1). The calls also presented harmonics and had a dominant frequency of between 80 and 130 Hz (Table 1).

**Table 1:** Summary of the main acoustic characteristics

	Pulse number	Pulse length (ms)	Pulse period (ms)	Call length (ms)
***Neoniphon sammara *Tulear**	**5 - 11**	**6.9 ± 0.1**	10.9 ± 2.4	110 - 150

***Neoniphon sammara *Rangiroa**	**4 - 9**	**5.9 ± 0.1**	8.5 ± 0.1	30 - 80

***Sargocentron diadema***	**6 - 11**	**6.8 ± 2**	14 ± 0.1	90 - 170

***Myripristis violacea***	6 - 11	**6.8 ± 0.2**	9 ± 0.1	40 - 110

***Myripristis kuntee***	4 - 9	**4.2 ± 0.1**	12.7 ± 0.1	40 - 110

***Holocentrus rufus***	4 - 6	4.1 ± 0.2	13.8 ± 0.3	45 - 80

Bold data: correlation with the fish size. These correlations were not tested in *Holocentrus rufus*.

### Neoniphon

**Figure 2:**
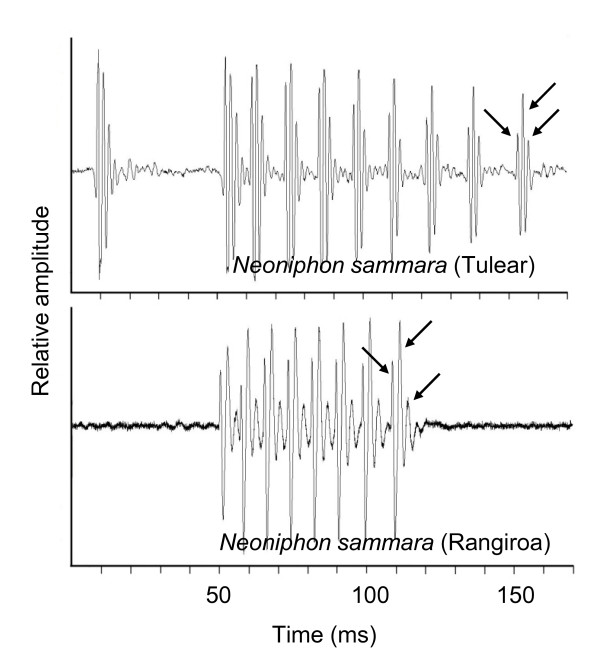
**Comparative oscillograms in two *Neoniphon sammara *populations**. Arrows indicate the peaks within the pulse.

In *Neoniphon sammara*, the different pulses of the train were made up of 3 peaks (Figure 2).

In Tulear, Madagascar, calls were characterised by a single pulse that was isolated at the beginning of the remaining train. This pulse was usually found at 42.5 ± 4 ms before the start of from the train. Trains were made up of 5 to 11 pulses, lasting from 110 to 150 ms. Pulse length was 6.9 ± 0.1 ms (N = 5, n = 1528) and pulse period was 10.9 ± 2.4 ms (N = 6, n = 1161).

In Rangiroa, calls did not show any isolated single pulses at the beginning of the call. Calls consisted of 4 to 9 pulses, lasting from 28 to 80 ms. Pulse length was 5.9 ± 0.1 ms (N = 11, n = 546) and pulse period was 8.5 ± 0.1 ms (N = 11, n = 455,).

In both populations, the number of pulses was significantly related to fish size: r = 0.35 (p < 0.001, N = 11, n = 111) in Rangiroa and r = 0.9 (p < 0.001, N = 5, n = 180) in Madagascar. We did not find any relationship between pulse period and fish size, allowing the comparison of periods from both populations. The pulse period was significantly longer in specimens from Madagascar than in those from Rangiroa.

In both populations, calls presented harmonics. There was no relationship between fish size and the fundamental frequency of the call. This was 109 ± 1 Hz (N = 6, n = 180) in Madagascar and 131 ± 1 Hz (N = 11, n = 111) in Rangiroa. Fundamental frequency was not found to be automatically the dominant frequency.

Settling larval *Neoniphon samara *were also recorded but it was not possible to extract these sounds from the background noise because they were too low.

### Sargocentron

**Figure 3:**
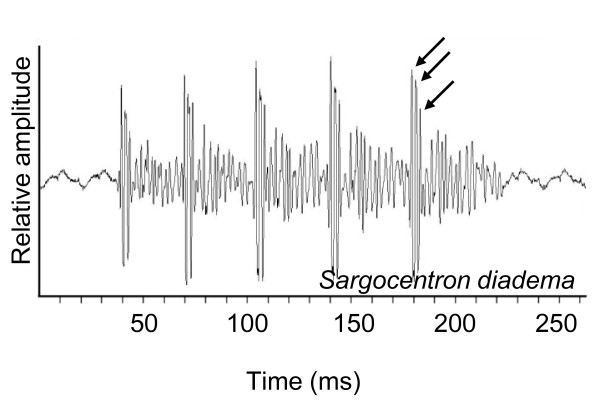
**Oscillogram in *Sargocentron diadema***. Arrows indicate the peaks within the pulse.

*In Sargocentron diadema*, the different pulses were also made up of three main peaks (Figure 3). The grunts were made up of 6 to 11 pulses, lasting from 92 to 170 ms (N = 5, n = 119) and the mean pulse duration was between 5 and 10 ms (X = 6.8 ± 2 ms, N = 5, n = 1290). This duration was significantly related to fish size (r = 0.54, p < 0.001, N = 5, n = 1290). The pulse period was 14 ± 0.1 ms (N = 5, n = 1037) and was not related to fish size. The fundamental frequency was 106 ± 1 Hz (N = 5, n = 150).

In Madagascar, sounds were recorded in one specimen of *Sargocentron spiniferum *(205 mm TL) but the amount of data was too small to carry out a correct analysis.

### Myripristis

**Figure 4:**
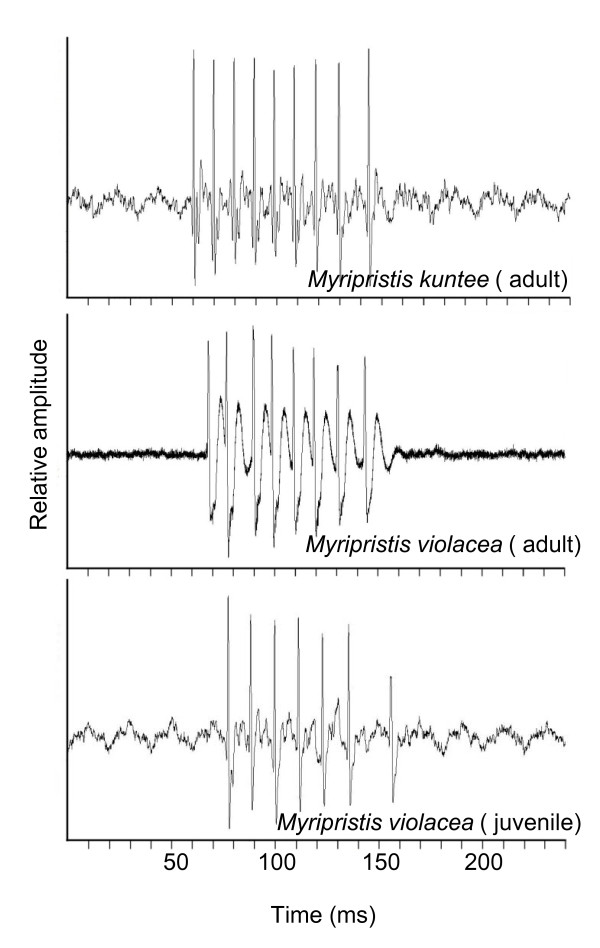
**Comparative oscillograms in different *Myripristis *populations**. Each pulse is supported by one main peak. *Myripristis kuntee *were recorded in Tulear and *Myripristis violacea *in Rangiroa.

In *Myripristis*, calls consisted of trains of pulses with each pulse having a single main peak (Figure 4). The pulse period tended to be longer towards the end of the calls (Figure 1).

In adult *Myripristis violacea*, calls were composed of 6 to 11 pulses (X = 7.6 ± 0.11, N = 11, n = 116). Sound length ranged from 40 and 110 ms and was correlated with the number of pulses (r = 0.74, p < 0.001, N = 11, n = 116). Weak but significant relationship was found between the number of pulses and fish size (r = 0.20, p = 0.02, N = 11, n = 116). Pulse length was 6.8 ± 0.2 ms (N = 9, n = 73) and related to the fish size (r = 0.7, p = 0.03, N = 9, n = 73).

**Figure 5:**
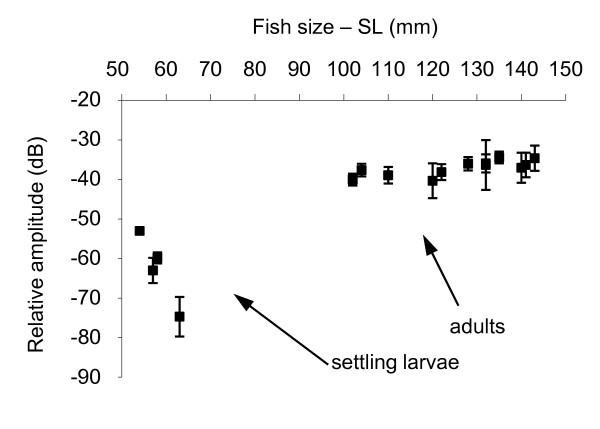
**Means of the sound level in *Myripristis violacea***.

In the five settling larvae with a size of between 57 mm and 62 mm, calls were made up of 4 to 8 pulses (X = 5.5 ± 0.25, n = 27), giving a sound length of between 30 and 78 ms. Sound length also appeared to be related to the number of pulses (r = 0.7, p < 0.001, N = 4, n = 27). The mean pulse period (Figure 5) was shorter (p < 0.001) in the settling larvae (X = 8.2 ± 0.1 ms, N = 5, n = 123) than in the adults (X = 9 ± 0.1 ms, n = 1075, N = 13). There was no significant relationship between pulse period and adult fish size (r = 0.09, p = 0.02, N = 12, n = 562). However, a relationship was found between pulse period and size when settling larvae were taken into account (r = 0.25, N = 12, n = 668). A relationship was also found between sound level (Figure 5) and fish size (r = 0.4, p < 0.001, N = 16, n = 146), resulting in a sound that was louder in adults (X = -37.2 ± 0.3 dB, N = 12, n = 123) than in settling larvae (X = -65.3 ± 1.3 dB, N = 4, n = 26).

**Figure 6:**
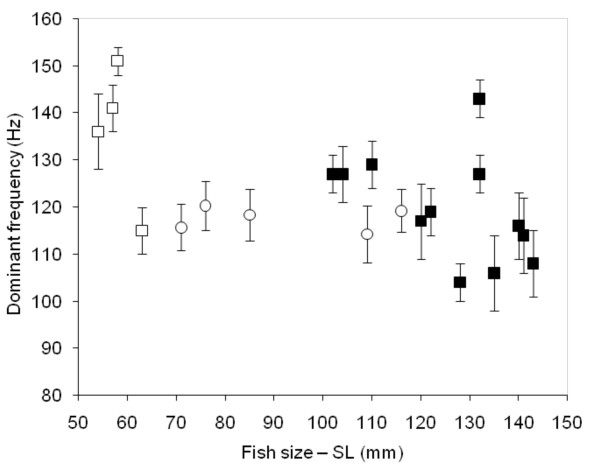
**Means of the fundamental frequencies in *Myripristis violacea *adults (■), in *M. violacea *larvae at the time of settlement (□) and in *Myripristis kuntee *(O)**. Note the fundamental frequency corresponds to the pulse period.

The calls presented harmonics. The frequency of the calls corresponded to the pulse period, and to fish size: smaller fishes showed higher frequencies (r = -0.5, p < 0.001, N = 16, n = 146). These statistical results do not, however, match the biology because some of the biggest fishes in Rangiroa showed the same fundamental frequency as the settling larvae (Figure 6).

**Figure 7:**
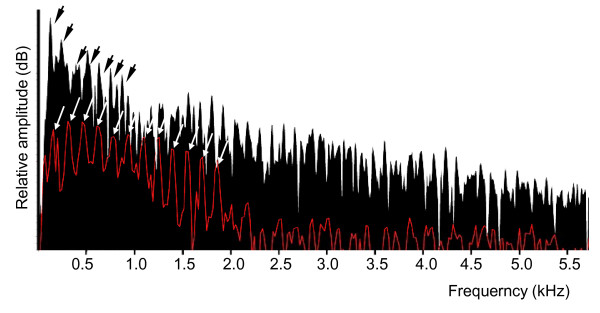
**Comparative power spectrum in calls of an adult (black) and a larva (red) of *Myripristis violacea***. Arrows indicate the different harmonics in each call. The sound pressure level appears less important in settling larvae and shows more harmonics. Power spectrum characteristics: sampling frequency 44.1 kHz, bandwith 31.5 Hz, hamming window.

The settling larvae also showed a different power spectrum from the adults. In settling fish, the number of harmonics is higher than in adults and some of them showed the same amplitude. The dominant frequency is not automatically the fundamental frequency. In adults, most of the energy was concentrated at the lower frequencies and there was also a constant lowering of the sound pressure level in the successive harmonics (Figure 7).

In *Myripristis kuntee*, sounds consisted of 4 to 9 pulses lasting between 40 and 110 ms. Pulse length was 4.2 ± 0.1 ms (N = 5, n = 369) and related to the fish size (r = 0.9, p < 0.001). The pulse period was on average statistically (p < 0.001) longer (X = 12.7 ± 0.1 ms, N = 5, n = 695) than in *Myripristis violacea *(X = 9 ± 0.1 ms, N = 13, n = 1075). In this species, we did not find any relationship between pulse period and fish size (r = 0.06, p = 0.11, N = 5, n = 695), or between sound level and fish size (r = 0.05, p = 0.51, N = 5, n = 150).

### Holocentrus

**Figure 8:**
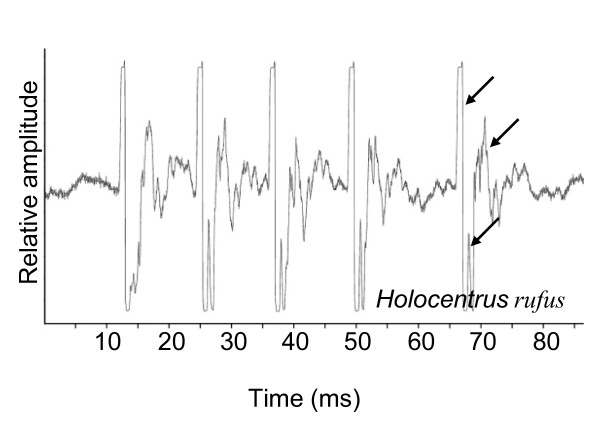
**Oscillogram in *Holocentrus rufus***. Arrows indicate the peaks within the pulse.

Sounds (Figure 8) in *Holocentrus rufus *consisted of calls of between four and six pulses, lasting between 45 and 81 ms. The pulse period was on average 13.8 ± 0.3 ms (N = 2, n = 84) and the pulse length was 4.1 ± 0.2 ms (N = 2, n = 49).

### Morphology

In all species, sounds produced by hand-held specimens produced vibrations felt at the level of the dorso-lateral region, behind the opercles. Dissections were performed at this level.

The morphology of the three species showed many common points. A detailed description is made of the morphology of *Sargocentron diadema*, which in many respects is representative of the other species of the study. Descriptions of differences between species are also noted *Sargocentron diadema*.

**Figure 9:**
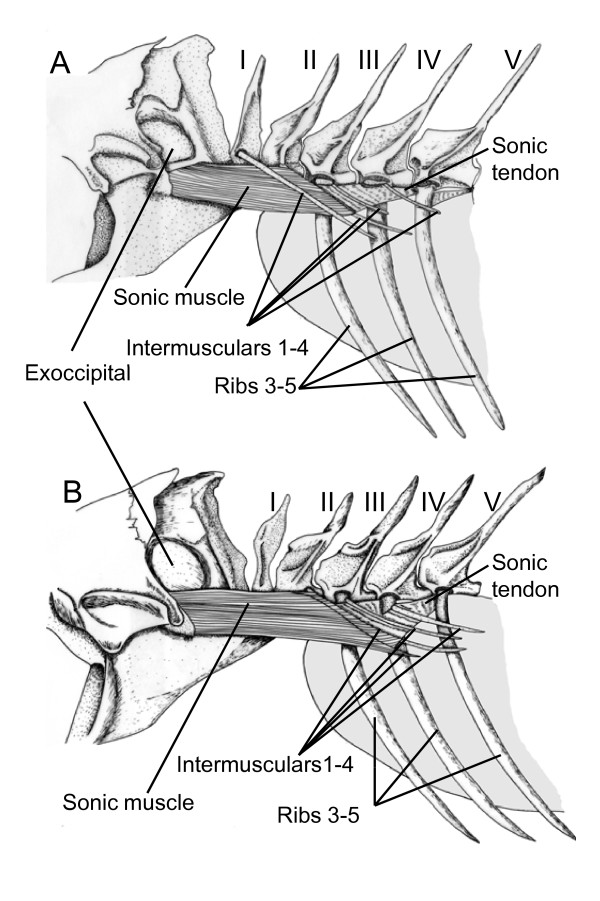
**Left lateral view of the sound producing apparatus in *Sargocentron diadema *(A) and in *Neoniphon sammara *(B)**. Latine numbers refer to the vertebra positions.

The sound-producing mechanism (Figure 9A) is found at the level of the first six vertebrae. Vertebrae I and II do not have ribs but each possess intermusculars that articulate at the level of the neural arch. Ribs articulate at the level of the vertebral body in vertebrae III to V, and on a short parapophysis on vertebra VI. In vertebrae III to VI, the intermuscular originate at the head of the ribs. Each rib possesses three ligaments. The first ligament inserts on the rostral part of the vertebral body and on the anterior face of the rib head. The second ligament is situated on the caudal part of the vertebral body and on the posterior face of the rib head. The third ligament is perpendicular to the vertebral body. It inserts on the middle part of the vertebral body, crosses ligament 2 ventrally and inserts on the mesial surface of the rib.

Sonic muscle originates on the neurocranium, at the level of the exoccipital, below an osseous protuberance corresponding to the area of the ductus semicircularis horizontalis of the inner ear. Caudally, sonic muscle runs below the first intermuscular bone and inserts mainly on the second intermuscular, and some muscle fibres are also inserted on the head of rib III. Ligaments (sonic ligaments) are found between intermusculars 2 and the proximal heads of ribs 4 and 5. Ventrally to these ligaments, there are also some fibres coming from the tendon of the sonic muscles but it is not easy to clearly differentiate the tendons and ligaments. These tendons also insert on the rib heads. A second ligament is situated between rib 5 and the short parapohysis of the sixth vertebra. Fibres from the hypaxial musculature also insert caudally on the heads of ribs 3 to 5.

The tunica externa of the anterior part of the swimbladder forms two small ligaments (SWB ligaments) inserting laterally on the enlarged head of the second intermuscular. Between the SWB ligaments, the antero-dorsal part of swimbladder shows a thinner zone, which seems to be deprived of tunica externa. This tissue inserts on the head margins of ribs 3 to 6, resulting in these heads being completely fused with the swimbladder and can be considered to be a part of it. This means that the back and forth movements of these articulated ribs involve simultaneously movements of the tunica externa of the swimbladder. There are also two thinner zones, which are situated fronto-laterally.

Sonic muscle contraction moves rostrally the heads of intermusculars 2, ribs 3 and the swimbladder ligaments. Because of the sonic ligaments, the heads of ribs 4 and 5 can also be displaced rostrally. There is no antagonist muscle. During muscle relaxation, the elasticity of ligaments, tendon and inner swimbladder pressure should help to restore the system.

### Neoniphon sammara

The sound-producing mechanism of *Neoniphon sammara *showed many similarities with *Sargocentron diadema *(Figure 9B). However, the sonic muscles possess fibres that insert on intermuscular 1.

### Holocentrus rufus

The sound-producing mechanism of *Holocentrus rufus *showed many similarities with *Sargocentron diadema*. The difference lies at the level of the swimbladder. We were not able to distinguish SWB ligaments inserting on the second intermuscular. The frontal part of the swimbladder was found to be in contact with the elongated auditory bulla, as described by Nelson (1955).

### Myripristis kuntee

**Figure 10:**
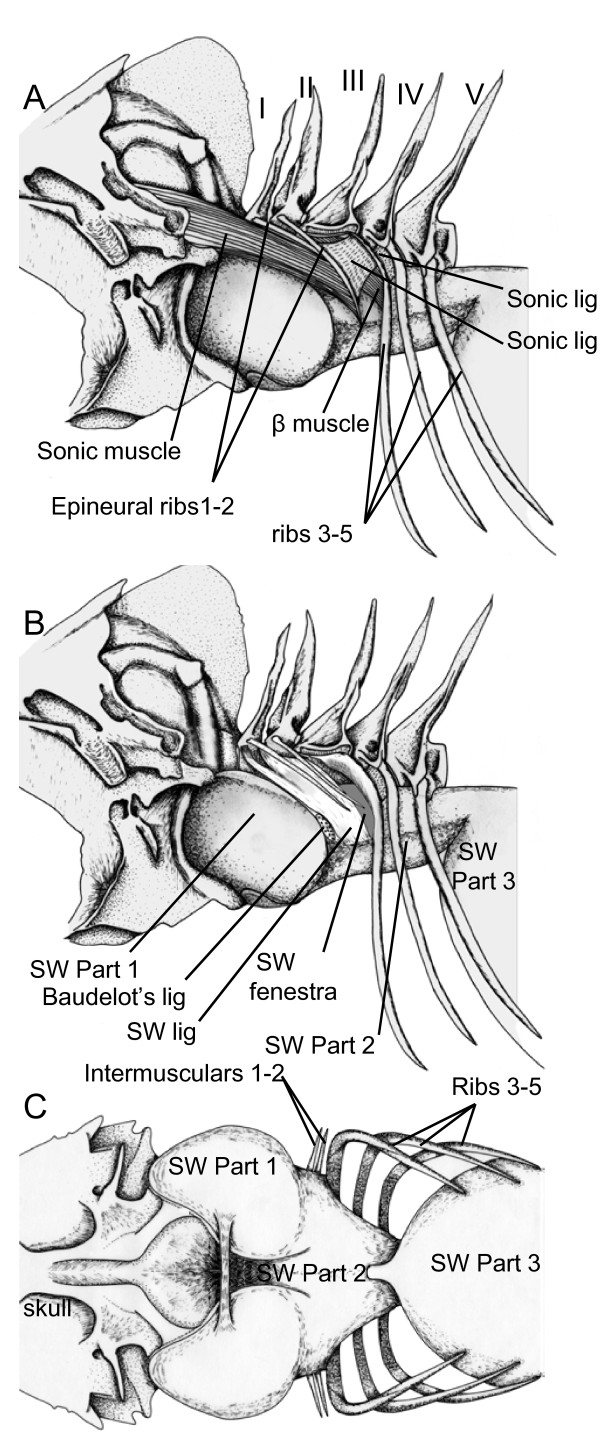
**Left lateral view of the complete (A) sound producing apparatus in *Myripristis kuntee***. Muscle and sonic ligaments were removed in (B) to show the swimbladder fenestra and swimbladder ligaments. In C, ventral view of the anterior part of the swimbladder, at the level of its association with the skull.

The sound-producing mechanism in *Myripristis *(Figure 10) showed many differences from *N. sammara *and *S. diadema*.

The five first vertebrae also possess two intermusculars on vertebrae I and II, and ribs on vertebrae III to V. On vertebrae I and II, the intermusculars originate high on the neural arch. The next three vertebrae possess ribs with intermusculars at the level of their head. The head of rib 3 is enlarged in the vertical plane and has a large ligament that holds it firmly to vertebrae III, preventing movement. This rib is the only bone that is closely associated with the tunica externa of the swimbladder, which insert on its posterior margin. The next ribs are placed above the swimbladder. Ribs 4 and 5 appear to have less freedom of movement because they are not articulated in sockets as in previously described species.

The sonic muscles are proportionally longer and larger than in previous species. The insertion of the sonic muscles on the skull is also more rostral on the exoccipital. Caudally the muscles insert on intermusculars 1 and 2. There is also a small muscle (β muscle) between intermuscular 2 and the proximal end of rib 3 but it is difficult to ascertain whether this muscle plays a role in sound production (Figure 10). *Myripristis *also shows two sonic ligaments: the first connects intermuscular 2 with rib 3, and the second connects rib 3 to rib 4.

Specialisations of the swimbladder are very particular [14]. The anterior part of the swimbladder forms two lateral projections, forming thick tubes in contact with the auditory bulla of the skull. Both tubes fuse caudally in a narrower channel running up to the sixth vertebra. The walls of this channel are thinner than the lateral projections. This second region is separated from the posterior chamber by a constriction.

Posterior to the swimbladder projections, the tunica externa develops anteriorly two kinds of flattened ligament, which insert on the proximal head of intermusculars 1 and 2, and on vertebrae I. The space between this ligament and rib 3 determines the swimbladder fenestra.

Contraction of the sonic muscles should pull anteriorly the first two intermusculars and associated swimbladder ligaments. This movement pulls anteriorly the rib 3 ligament and consequently moves rib 3. Due to ligaments 1 and 2, rib 3 should, however, have restrained anterior movements.

### Electron microscopy

**Figure 11:**
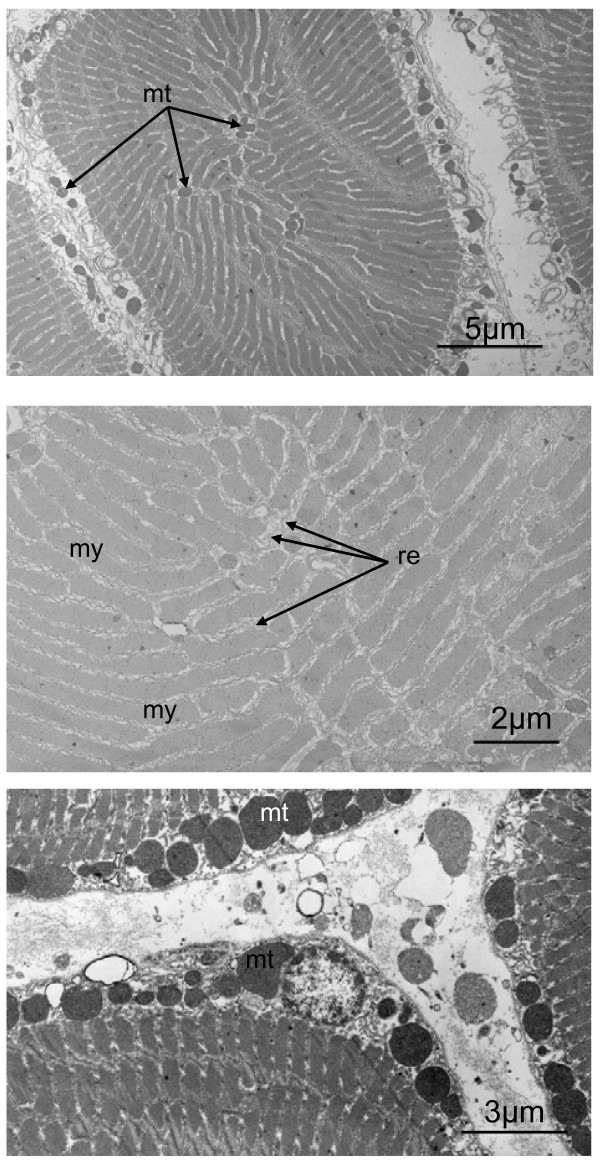
**Different TEM pictures of transverse sections in sound producing muscle in *Myripristis***. These cells are characterized by their small sections, the high number of mitochondria in periphery and the well developped reticulum sarcoplasmic. mt: mitochondria; my: myofibril; re: reticulum sarcoplasmic.

Results from transmission electron microscopy were similar between the species. In comparison to their white epaxial muscles, sonic muscles (Figure 11) in the studied holocentrids present the following characteristics: the sonic muscles are more innervated and more irrigated and the diameter of their fibres and myofibrils diameters are smaller; their mitochondria are more numerous and situated in the periphery, under the sarcolem and close to the blood capillary; and their reticulum sarcoplasmic is more developed in the sonic muscles.

## Discussion

Different kinds of sounds (staccatos, grunts, knocks, thumps, growls) have been described in some Holocentridae [3,4,6-8,15]. However, this nomenclature is not reliable because the description of a sound is not always based on physical parameters. Consequently, some names are redundant and could apply to the same kind of sound. The "pops" and "pop volleys" that were described by Bright and Sartori [15] could correspond to Salmon's [6] "knocks" and "staccatos". Also, some authors have advocated that grunts are made only by fish while they are hand-held, stressing that the presence of these sounds has not been detected in the environment. However, Horch and Salmon [7] noted that growls were the usual response made by a field population of *M. violaceus *to disturbances caused by a diver, whereas other sounds (thumps and knocks) were produced between conspecifics. These "growls" consisted of many pulses in rapid succession with a consistent tendency toward slower pulse repetition rates at the end of the sound. This signature corresponds to the hand-held sounds (grunts) we observed in all species during our study.

In the gourami [16], damselfish [17-19], anemonefish [20] and pearlfish [21], dominant frequency has been shown to decrease in larger fishes. In these examples, the high slope value of the correlation between fish size and dominant frequency indicates that the size of the emitter can be assessed by the receiver and so be used in sonic communication. In the grunt of the gurnard *Eutrigla gurnardus *[22], in the weakfish *Cynoscion regalis *[23], in the toadfish *Halobatrachus didactylus *[24] and in the holocentrids of this study, this kind of relationship has also been statistically established. However, the slope value is very weak and it is difficult to determine whether the fish can discriminate the spectral characteristic of the call as in the previous group. This hypothesis is well supported by recent studies on the toadfish in which the pulse period (and consequently the call frequency) can be related to the male quality [25,26] but not to its size. Calling fishes are classically divided into categories on the basis of their sound-producing apparatus. Another way to categorize sound production would be to base it on the kind of information the fish is able to communicate. In fishes with a sound-producing mechanism based on the fast-contracting muscles (holocentrids, sciaenids, batrachoidids), size could not be inferred from the pulse period. According to the species, this pulse period may correspond to the fundamental frequency. In *M. violacea*, for example, fish of 60 mm and 130 mm can have the same frequency (Figure 2). As a comparison, the calling frequency of 60 mm clownfish is 700 Hz, whereas it is less than 400 Hz in 130 mm specimens [20]. Calling amplitude could be used to identify the difference between settling larvae and adults but its period homogeneity would not help to discriminate the adult fish from the larvae. Pulse duration was statistically related to the fish size in all species. The biggest differences between the pulse duration of smallest (57 mm SL) and largest fish (143 mm SL) was found in *M. Violacea*, and was < 5 ms. Once more, it means the slope value is very weak and it is difficult to determine whether the fish can use these temporal differences.

In grunts made by hand-held *Holocentrus rufus *[8] and in the grunts in this study, the calls were made up of pulses with the same rate range, between 90 and 120 Hz. In *Holocentrus rufus*, the sound-producing mechanism depends on the contraction of the bilateral pair of extrinsic muscle whose contraction rates correspond to the fundamental frequencies [8,9]. As *Myripristis*, *Sargocentron *and *Neoniphon *produce grunts with the same kind of pulse rate and roughly the same type of sound- producing apparatus as in *H. rufus*, it can reasonably be inferred that the mechanism of sound production is similar in all these genera.

In groups with extrinsic sonic muscles inserting on a precise point of the swimbladder, modifications of the bladder wall (e.g. sclerification, ossification) or of the tendinous insertions on the swimbladder are possibly a response to the mechanical stress created by contraction of the sonic muscles [27]. In other fish with fast-contracting muscle units, the following observations have been made regarding the muscles 1) they are directly inserted on areas covering important parts of the swimbladder [28,29], or 2) they possess tendons running from the left to the right sonic muscle, as in piranhas [30] or 3) they lie on the body wall and extend along almost the entire length of the swimbladder, where fibres insert at the level of a dorsal aponeurotic sheet [31]. The osseous ribs on which the muscles insert in Holocentridae seem to be a required intermediary to avoid any mechanical stress or damage to the swimbladder walls. Moreover, our morphological study here highlighted the fact that the first articulated ribs are closely integrated into the swimbladder. Therefore, the sound cannot result from the direct vibration of the swimbladder wall. Each sonic muscle contraction leads to a rostral displacement of the proximal end of the first ribs and of the anterior part of the swimbladder, the posterior swimbladder part being incapable of displacement. The displacement is, however, brief because of the numerous ligaments between the vertebrae and the ribs. The abrupt arrest in the displacement in addition to the fast acting muscle could originate the short pulses of the call. The sound-producing muscle ultrastructure reinforces the proposed mechanism. As in many fishes with fast twitch contractions, the fibres are narrower than in white epaxial fibres, the cells consist of alternating ribbons of sarcoplasmic reticulum and myofibrils, numerous mitochondria are found in periphery, and the sarcoplasmic reticulum is well developed. All these characteristics are well known in other fishes with fast-contracting muscles [31-34].

**Figure 12:**
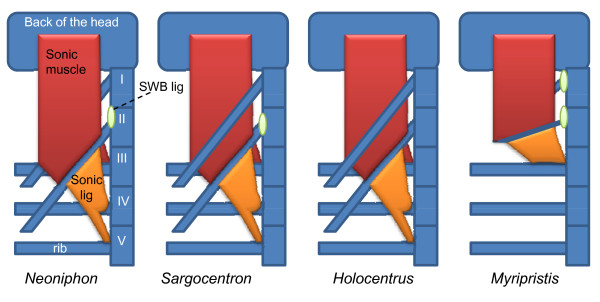
**Schematic dorsal view of the left part of the sound producing apparatus in different Holocentridae species**.

The main difference between the sound-producing mechanism in the Myripristinae and Holocentrinae of this study lies in the sonic muscle tendons and the number of ribs involved (Figure 12). In the *Myripristis *species, the pulse is composed of one peak [5,6], which could correspond to the movement of rib 3. In *Neoniphon *and *Sargocentron*, the pulse has been typically found to possess three peaks, which could correspond to the movement of three ribs. It could also explain the pulse duration is related to fish size in *Neoniphon *and *Sargocentron*, but not in both *Myripristis *species. In the *Holocentrus rufus *of our study, and in the work of Winn and Marshall [8] and Fish and Mowbray [5], the pulse seems mainly to be made up of at least two peaks, maybe three. The situation is however less clear than in previously studied Holocentrinae, and this observation needs to be confirmed in the future through the recording of additional species.

Winn and Marshall [8] showed in *Holocentrus rufus *that the time between the penultimate and the final pulse was always greater than the time between earlier pulses. Our analyses of grunts showed that the same phenomenon occurred in *Myripristis*, *Sargocentron *and *Neoniphon*. However, we went further and showed that there was a constant increase in the pulse period during the call (Figure 1). The lengthening of the pulse period could be associated to muscle fatigue. Generally speaking, muscle fatigue seems to imply a decrease in the amplitude of the pulses [35,36] but not a lengthening of the period between contractions. According to Jones et al. [37], a decline in discharge rate could help to optimise force production: due to the peripheral fatigue-processes, less total force is produced by a steady high-frequency discharge than by a discharge of decreasing rate. The contractions maintained were, however, much longer in the experiments of Jones et al. and involved more contractions than in Holocentridae.

The present study did not reveal information regarding only the sound-producing mechanism in Holocentridae. It also showed for the first time that *Myripristis *and *Neoniphon *larvae are able to make sounds as soon as they settle on the reef. Except for an obvious difference in size, we did not notice any difference in the sound-producing mechanism between settling larvae and adults. Lo-Yat [38] found that the duration of the planktonic larval stage in the Rangiroa holocentrids he studied lasted between 40 and 65 days. In *Myripristis*, we showed the number of pulses and their amplitudes were lower in the larvae than in the adults. Although the mechanisms are not the same, these results are in concordance with studies involving fishes from other taxa: the croaking gourami *T. vittata *[16,39] and the gurnard *E. gurnardus *[22].

Dialects have already been established in different fish species [40-43]. In these examples, differences in sound production have been found to be mainly the result of physiology. This is also the case in *Neoniphon sammara*, in which one of the differences corresponded to the pulse period. However, both populations showed an intriguing difference at the level of the "phraseology": we found an isolated pulse at the beginning of the call in Madagascar whereas this was not the case in French Polynesia (Figure 2). This observation raises many questions because sounds were made when fish were hand-held. Deeper ethological observations are needed to know what sounds are made in natural conditions.

Ideally, the findings of this study should be taken further using data from other holocentrid species. These fish are very easy to record when hand-held and a detailed comparative study of their morphology would undoubtedly increase our understanding of how this complex sound-producing organ has evolved. Despite the fact that all Holocentrinae have a roughly similar sound-producing mechanism (Figure 12), further research would enable us to better understand the differences that nevertheless exist between them in this regard.

## Conclusion

It is doubtful this family can use the fundamental frequencies (or pulse periods) to infer the size of partner. Pulse duration and number of pulses are statistically related to fish size. However, it is doubtful these characteristics are used because the slope values are weak, giving few differences between calls within the species. However, it remains other features (sound amplitude, resistance to muscle fatigue, calling frequency) could be used to assess the body size. Characteristics of the sound producing mechanisms are conservative. All species possess however fast-contracting muscles and have the same kind of sound producing mechanism. They do show some change between clades but these differences are not important enough to deeply modify the waveforms of the calls. In this case, our description of the grunt could be considered as the signature for the holocentrid family and can be used in passive acoustic monitoring.

## Competing interests

The authors declare that they have no competing interests.

## Authors' contributions

EP conceived of the study, designed the experiments, analyzed acoustic sequences from Rangiroa and wrote the paper. PV, CB and DL collected the fish and participated to the fish recordings. LD recorded the fish and analyzed acoustic sequences from Madagascar. All authors read and approved the final manuscript.
